# Therapeutic Role of miR-30a in Lipoteichoic Acid-Induced Endometritis via Targeting the MyD88/Nox2/ROS Signaling

**DOI:** 10.1155/2021/5042048

**Published:** 2021-12-31

**Authors:** Kangfeng Jiang, Weiqi Ye, Qian Bai, Jinyin Cai, Haichong Wu, Xiaobing Li

**Affiliations:** ^1^College of Veterinary Medicine, Yunnan Agricultural University, Kunming, 650201 Yunnan, China; ^2^Fujian Provincial Key Laboratory for Prevention and Control of Animal Infectious Diseases and Biotechnology, Longyan 364012, China; ^3^Department of Veterinary Medicine, College of Animal Sciences, Zhejiang University, Hangzhou, 310058 Zhejiang, China

## Abstract

*Staphylococcus aureus* (*S. aureus*), a notorious pathogenic bacterium prevalent in the environment, causes a wide range of inflammatory diseases such as endometritis. Endometritis is an inflammatory disease in humans and mammals, which prolongs uterine involution and causes great economic losses. MiR-30a plays an importan trole in the process of inflammation; however, the regulatory role of miR-30a in endometritis is still unknown. Here, we first noticed that there was an increased level of miR-30a in uterine samples of cows with endometritis. And then, bovine endometrial epithelial (BEND) cells stimulated with the virulence factor lipoteichoic acid (LTA) from *S. aureus* were used as an in vitro endometritis model to explore the potential role of miR-30a in the pathogenesis of endometritis. Our data showed that the induction of the miR-30a expression is dependent on NF-*κ*B activation, and its overexpression significantly decreased the levels of IL-1*β* and IL-6. Furthermore, we observed that the overexpression of miR-30a inhibited its translation by binding to 3′−UTR of MyD88 mRNA, thus preventing the activation of Nox2 and NF-*κ*B and ROS accumulation. Meanwhile, in vivo studies further revealed that upregulation of miR-30a using chemically synthesized agomirs alleviates the inflammatory conditions in an experimental mouse model of endometritis, as indicated by inhibition of ROS and NF-*κ*B. Taken together, these findings highlight that miR-30a can attenuate LTA-elicited oxidative stress and inflammatory responses through the MyD88/Nox2/ROS/NF-*κ*B pathway and may aid the future development of novel therapies for inflammatory diseases caused by *S. aureus*, including endometritis.

## 1. Introduction

Endometritis is a reproductive disorder characterized by local inflammation of the endometrium, which leads to delayed uterine involution and huge economic losses [1–3].Gram-positive bacteria *Staphylococcus aureus* (*S. aureus*) has been recognized as a typical opportunistic pathogen prevalent in the environment, which has the potential to induce endometritis [4]. *S. aureus* is highly resistant to multiple antibiotics, subsequently making treatment more difficult [5].Therefore, it would be highly desirable to discover and develop novel therapeutic approaches to endometritis caused by *S. aureus*.

Lipoteichoic acid (LTA), a negatively charged glycolipid, exists on the cell wall surface of *S. aureus* [6]. Emerging evidence has shown that LTA has the same proinflammatory properties as lipopolysaccharide (LPS) and plays a crucial role in the inflammatory reactions induced by *S. aureus* [7, 8]. Endometrial epithelial cells express toll-like receptors (TLRs) and participate in the pathogenesis of endometritis [9]. TLRs recognize pathogen-associated molecular patterns (PAMPs) of pathogenic bacteria and then trigger inflammatory responses via intracellular signaling cascade, in which MyD88 is an important adaptor protein [10, 11]. More importantly, a recent report using purified LTA from *S. aureus* has clearly indicated that *S. aureus* LTA can efficiently stimulate monocytes through TLR2to secrete inflammatory mediators [12].

When cells are subjected to oxidative stress elicited by inflammatory responses, upregulated levels of reactive oxygen species (ROS) may produce various deleterious effects within the tissues [13]. It has previously been observed that ROS are implicated in the process of many inflammatory diseases, including endometritis [14]. NADPH oxidase is considered to be an important source of ROS under a variety of pathologic conditions. In recent studies, Lee et al. have reported that LTA induces ROS production via the MyD88-mediated NADPH oxidase2 (Nox2) pathway [15]. High intracellular level of ROS can activate downstream signaling pathways, including NF-*κ*B pathway [16]. NF-*κ*B is a key transcription factor involved in a diverse set of pathological processes, such as bacterial-induced inflammation. Once activated, NF-*κ*B promotes the transcription of inflammatory genes and subsequent the secrete of inflammatory cytokines, further aggravating cell and tissue damage [17].Thereafter, blockage of ROS-mediated NF-*κ*B activation can be an effective treatment method to improve the outcome of inflammatory diseases such as endometritis.

In the last decades, the regulatory function of microRNAs (miRNAs) in various biological processes has attracted extensive attention. MiRNAs are an evolutionarily conserved class of small noncoding RNA, which negatively modulate the gene expression predominantly through binding to 3′-untranslated region (UTR) [18]. A considerable number of studies on the regulatory effects of miRNAs have revealed the relationship between miRNAs and inflammation. Indeed, abnormal expression levels of miRNAs are closely associated with the occurrence of various inflammatory diseases, such as asthma [19], atherosclerosis [20], and pneumonia [21]. Numerous miRNAs have been confirmed to restrain NF-*κ*B pathway via inhibiting NF-*κ*B-activating proteins. For instance, miR-16and miR-223 blunt NF-*κ*B pathway by downregulating IKK*α* [22]. Notably, studies that determine the molecular modulator of endometritis have implied the involvement of miRNAs in the pathogenesis of endometritis [23]. Our previous study has also shown that upregulation of the miR-92b expression improves the outcome of endometritis in mice via targeting PTEN [17].miR-30a is a highly conserved and multifunctional miRNA and has been demonstrated to be involved in tumor growth and immune response. It was found that miR-30a regulates IRF4 to influence IL-17-associated autoimmune inflammation and also mediates the inflammatory macrophage polarization [24]. In particular, upregulation of miR-30a targets Neurod1 and alleviates the inflammatory responses during injured spinal cord [25]. However, the regulatory function of miR-30a in *S. aureus* endometritis has not been studied. In this study, we screened the possible targets of miR-30a using the online miRNA target prediction program and identified MyD88 as a potential target ofmiR-30a. In addition, we have also determined that the miR-30a overexpression impairs the inflammatory response induced by *S. aureus* LTA stimulation. Thus, miR-30a may be a new therapeutic target for inflammatory diseases such as *S. aureus* endometritis.

## 2. Materials and Methods

### 2.1. Collection of Uterine Tissue Samples

Uterine tissues from cows with and without endometritis (*n* = 3) were collected according to the guidelines and stored at -80°C for further experiments.

### 2.2. Mouse Model and Sampling

BALB/c mice (female, 6-8 weeks old) were provided by the Experimental Animal Center of Zhejiang University (Hangzhou, China). The mice were randomly assigned into four groups (*n* = 10): agomir-NC group, agomir-30a group, agomir-NC+ LTA group, and agomir-30a + LTA group. An experimental mouse model of endometritis was established as previously described [14]. The mice were perfused equal amounts of LTA (1 mg/kg) on each side of the uterus to induce endometritis, and the control group received equal volumes of PBS. In order to increase the expression of miR-30a, the mice were injected with chemically synthesized agonists (agomir-30a, 0.5 *μ*mol/kg) for three consecutive days before LTA administration. Then, the mice were euthanized at 24 h after LTA administration, and the uterine tissue from each group was collected for molecular biological analysis. All experiments were repeated three times. All animal experiments involved in the research were approved by the Ethical Committee on Animal Research at Yunnan Agricultural University (Kunming, China).

### 2.3. Histopathological Analysis

The tissue samples were fixed in 4% paraformaldehyde, dehydrated with graded alcohol, and embedded in paraffin. And then, the samples were sectioned and stained with hematoxylin and eosin (H&E). The histopathological abnormalities were assessed under an optical microscope (Olympus, Japan).

### 2.4. Cell Culture

BEND cells were cultured in Dulbecco's Modified Eagle's Medium (DMEM, HyClone, USA) containing 10% FBS at 37°C with 5% CO_2_. After the cell state is stable, the cells were treated with LTA (10 *μ*g/mL) alone or with other treatments. After the indicated treatments, the cells were collected for further experiments.

### 2.5. Cell Transfection

The mimics, inhibitors, siRNAs, and their negative controls (NC) were synthesized by GenePharma (Shanghai, China). Cells were transfected with mimics, inhibitors, or siRNAs with Lipofectamine 2000 (Invitrogen, USA) following the manufacturer's instructions.

### 2.6. QPCR Analysis

Total RNA from tissues or cells was extracted with TRIzol Reagent. The concentration of total RNA was determined by the spectrophotometer Q5000 (Quawell Technology, USA) and then reverse-transcribed into cDNA by using a reverse transcription kit (Vazyme Biotech, China). qPCR was carried out using a real-time PCR kit (GenePharma, China) in accordance with the manufacturer's instructions. The 2^–*ΔΔ*Ct^ method was utilized to calculate the relative expression of genes. MiR-30a was normalized to U6 snRNA, and the IL-1*β* and IL-6 expression was normalized to GAPDH. The primers are listed in Supplementary Table 1.

### 2.7. Enzyme-Linked Immunosorbent Assay (ELISA)

The amounts of IL-1*β* and IL-6 were detected by ELISA kits (BioLegend, Camino Santa Fe, USA) following the manufacturer's instructions.

### 2.8. Preparation of Protein Extracts and Western Blot Analysis

The protein extracts were prepared with ice-cold RIPA lysis buffer (Beyotime, China). After quantitative determination of protein concentration in each sample with a BCA assay kit, equal amounts of protein were subjected to 10% SDS-PAGE and then electrotransferred onto polyvinylidene fluoride (PVDF) membranes, which was blocked with 5% nonfat milk at room temperature for 2 h. Then, the membranes were probed with primary antibodies for overnight at 4°C, followed by incubation with secondary antibodies for 1 h at room temperature. The quantification of the protein expression was normalized to *β*-actin using the ImageQuant LAS 4000 mini (GE Healthcare).

### 2.9. Luciferase Reporter Assay

The possible binding site between miR-30a and MyD88was predicted using TargetScan 7.2. To assess whether MyD88 is a direct target of miR-30a, we cloned 3′-UTR of MyD88 into a psiCHECK™-2 vector to generate the luciferase reporter vectors containing wild or mutant 3′-UTR of MyD88. For the luciferase reporter assay, HEK293T cells were cotransfected with the luciferase reporter vectors and miR-30a mimics/NC. After 24 h of transfection, the cell lysates were harvested with passive lysis buffer, and the luciferase activities were then detected with a dual-luciferase assay kit (Promega, USA).

### 2.10. Intracellular ROS Assay

The intracellular ROS were measured using the fluorescent probe 2′,7′-dichlorofluorescein-diacetate (DCFH-DA). After the corresponding treatment, BEND cells were incubated with 10 *μ*M DCFH-DA in the dark at 37°C for 30 min. The cells were washed twice with PBS, and then the fluorescence intensity was assayed with a fluorescence microscope (Leica,Germany).

### 2.11. Immunofluorescence Assay

After the corresponding treatment, the cells were collected and fixed with 4% paraformaldehyde, permeabilized for10 min with 0.1% Triton X-100, and then blocked with 3% bovine serum albumin (BSA) for 1 h. Then, the cells were washed and incubated with special primary antibodies overnight at 4°C. Next, the cells were incubated with a fluorescent-labeled second antibody for 1 h at room temperature. The cell nuclei were stained using DAPI for 15 min in the dark, and the fluorescence images were visualized using a fluorescence microscope (Olympus,Japan).

### 2.12. Statistical Analysis

Data were calculated using the GraphPad Prism Program (GraphPad, USA) and expressed as mean ± SEM. Differences between two groups were analyzed using an unpaired Student's *t*-test, and one-way analysis of variance (ANOVA) was used for multiple comparisons. A value of *P* < 0.05 or 0.0.1 was considered statistically significant.

## 3. Results

### 3.1. Increased miR-30a Expression Is Associated With Endometritis

**Figure 1:**
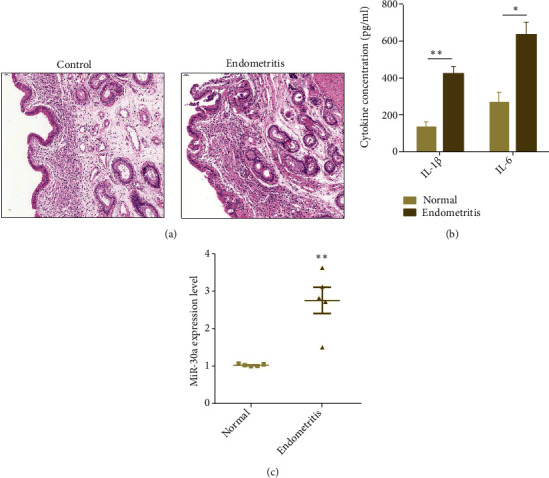
Expression of miR-30a in endometritis. (a) Analysis of pathohistology. Scale bar = 50 *μ*m. (b) The levels of IL-1*β* and IL-6 were measured by ELISA. (c) The miR-30a expression was detected by qPCR. Data are presented as mean ± SEM. ^∗^*P* < 0.05, ^∗∗^*P* < 0.01.

As displayed in Figure 1(a), hyperemia, edema, cell necrosis, and inflammatory cells were observed in the endometritis group when compared with the normal group. Some key inflammatory mediators have been found to be related to the occurrence and development of endometritis, including IL-1*β* and IL-6 [26].Thus, the expression levels of IL-1*β* and IL-6 were measured. ELISA results showed that these proinflammatory cytokines were remarkably increased in the endometritis group (Figure 1(b)). In addition, the miR-30a level was also notably upregulated in the endometritis group when compared to the normal group (Figure 1(c)). These data reveal that upregulated miR-30a might be associated with endometritis.

### 3.2. Elevated Expression of miR-30a following *S. aureus* LTA Stimulation

**Figure 2:**
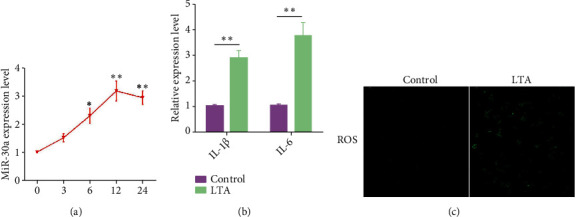
LTA increases the miR-30a expression in BEND cells. (a) BEND cells were administrated with 10 *μ*g/mL LTA at different times, and the miR-30a expression was assessed by qPCR. (b) BEND cells were administrated with 10 *μ*g/mL LTA for 12 h, and the release of IL-1*β* and IL-6 was detected by qPCR. (c) The ROS production was determined by means of the fluorescent probe DCFH-DA. Scale bar = 50 *μ*m. Data are presented as mean ± SEM. ^∗^*P* < 0.05, ^∗∗^*P* < 0.05.

Bovine endometrial epithelial (BEND) cells were treated with *S. aureus* LTA for different times, and the miR-30a expression was determined by qPCR. As shown in Figure 2(a), the miR-30a expression was increased in a time-dependent manner upon LTA stimulation and reached a peak at 12 h. Besides, the IL-1*β*and IL-6 levels were also notably increased at 12 h (Figure 2(b)),accompanied by an elevated level of ROS(Figure 2(c)).These results suggest that the upregulatedmiR-30a expression was induced by LTA stimulation in BEND cells.

### 3.3. NF-*κ*B-Dependent Induction of miR-30a upon *S. aureus* LTA Stimulation

**Figure 3:**
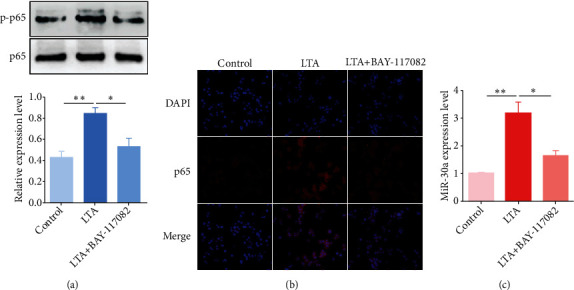
NF-*κ*B activation promotes the induction of miR-30a. (a) BEND cells were pretreated with BAY-117082 for 1 h and then exposed to LTA for 12 h. Western blot was performed to detect the p-p65 and p65 protein levels. The relative expression level of p-p65 was normalized to p65. (b) Nuclear translocation of NF-*κ*B p65 was analyzed by immunofluorescence staining. Scale bar = 20 *μ*m. (c) The miR-30a expression was detected by qPCR. Data are presented as mean ± SEM. ^∗^*P* < 0.05, ^∗∗^*P* < 0.01.

NF-*κ*B is an essential regulator of the inflammatory response during endometritis [17].Our results indicated that the phosphorylatedNF-*κ*B p65 (p-p65) level was obviously increased following *S. aureus* LTA stimulation (Figure 3(a)).Recent studies have reported that NF-*κ*B is able to bind to the promoter region of miR-30 to induce its transactivation [27]. To determine whether the upregulation of miR-30a by LTA was also mediated by NF-*κ*B activation, we constrained NF-*κ*B activities by means of a specific NF-*κ*B inhibitor (BAY-117082). As expected, treatment with BAY-117082 significantly blocked the nuclear translocation of p65, which was consistent with the western blot analysis of p-p65(Figures 3(a) and 3(b)). Moreover, the miR-30aexpression induced by LTA was also obviously decreased after NF-*κ*B inhibition (Figure 3(c)). These results imply that induction of the miR-30a expression by LTA depends on NF-*κ*B activation.

### 3.4. Overexpressionof miR-30a Impairs the Inflammatory Cytokine Production

**Figure 4:**
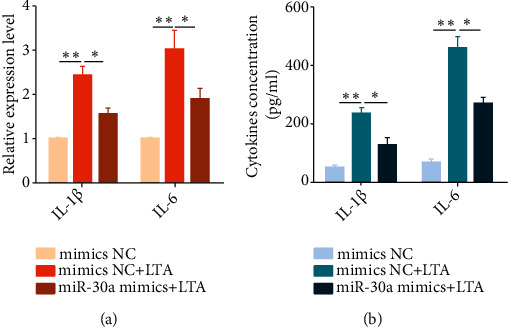
Overexpressing miR-30a reduces LTA-induced IL-1*β* and IL-6 production. BEND cells were transfected with miR-30a mimics for 24 h and exposed to LTA for 12 h. The proinflammatory cytokines IL-1*β* and IL-6 were determined by qPCR (a) and ELISA (b). Data are presented as mean ± SEM. ^∗^*P* < 0.05, ^∗∗^*P* < 0.01.

LTA can activate NF-*κ*B and then promote the release of proinflammatory cytokines, which promote the development of endometritis [14]. To uncover the potential role ofmiR-30a in proinflammatory cytokine production, BEND cells were transfected with miR-30a mimics and then exposed to LTA for 12 h. As displayed in Figures 4(a) and 4(b), the overexpression of miR-30a remarkably decreased LTA-induced IL-1*β* and IL-6 secretion. These data demonstrated that miR-30a plays an anti-inflammatory role in the LTA-induced inflammatory reactions.

### 3.5. MyD88 Is a Molecular Target of miR-30a

**Figure 5:**
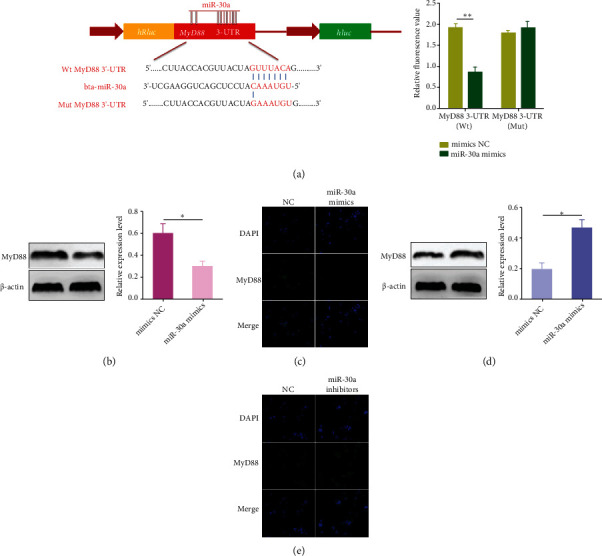
MyD88 is a molecular target of miR-30a. (a) The alignment of miR-30a and MyD88 3′-UTR by computational prediction. HEK293T cells were cotransfected with the MyD88 3′-UTR luciferase reporter vectors and miR-30a mimics/NC. And then, the dual-luciferase reporter assay was carried out. (b, d) BEND cells were transfected with miR-30a mimics or inhibitors for 24 h, and the MyD88 expression was detected by Western blot. (c, e) Immunofluorescence staining of MyD88. Scale bar = 20 *μ*m. Data are presented as mean ± SEM. ^∗^*P* < 0.05, ^∗∗^*P* < 0.01.

To unveil the potential mechanisms through which miR-30a exerts its anti-inflammatory activity, its potential molecular targets were predicted. Among all the putative targets, we selected MyD88 related to the TLRs/NF-*κ*B signaling as the target for miR-30a in the present study. The putative target seed sequence was shown in Figure 5(a). To further ensure that miR-30a is capable of targeting MyD88, a luciferase reporter assay was performed. As shown in Figure 5(a), the introduction of miR-30a was able to markedly inhibit the luciferase activity of HEK293T cells transfected with the luciferase reporter vectors containing wild 3′-UTR of MyD88, but not those transfected with the mutated vectors. Furthermore, the MyD88 protein was detected in BEND cells treated with miR-30a mimics or inhibitors. We found that the overexpression of miR-30a markedly decreased the MyD88 expression (Figures 5(b) and 5(c)), while inhibition of miR-30a increased the level of MyD88 (Figures 5(d) and 5(e)). Overall, these results suggest that MyD88 is a molecular target of miR-30a.

### 3.6. miR-30a Dampened NF-*κ*B Activation by Inhibiting the MyD88/Nox2/ROS Axis

**Figure 6:**
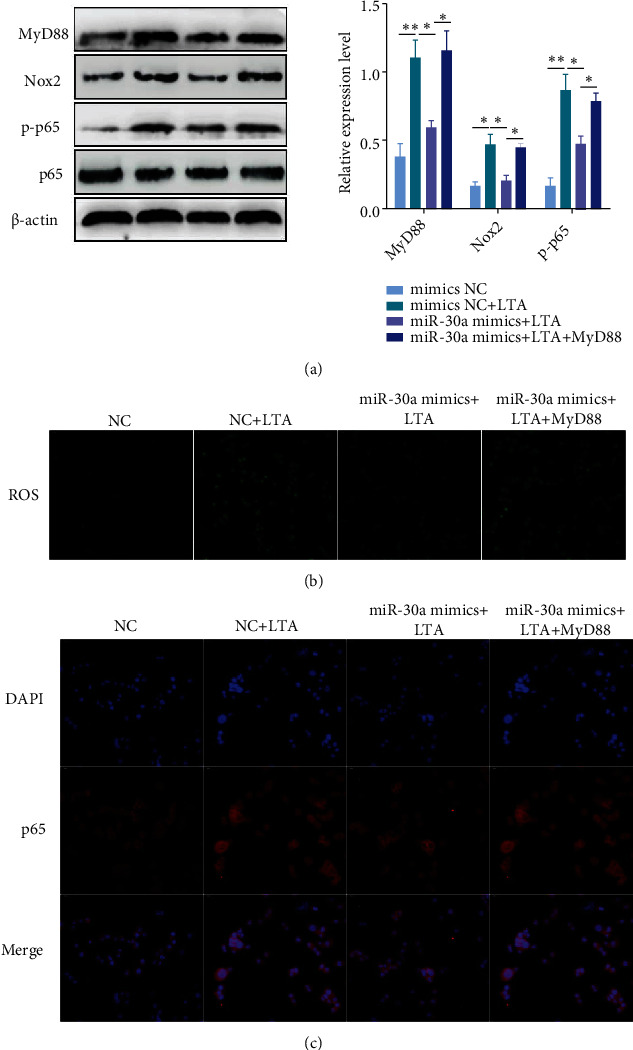
Overexpressing miR-30a prevents LTA-induced NF-*κ*B activation by inhibiting the MyD88/Nox2/ROS axis. (a) BEND cells were cotransfected with miR-30a mimic and pcDNA3.1-MyD88 for 24 h, followed by 12 h of exposure to LTA. Then, the levels of MyD88, Nox2, p-p65, and p65 were measured by Western blot. (b) The levels of ROS were detected using the fluorescent probe DCFH-DA. Scale bar = 20 *μ*m. (c) Nuclear translocation of NF-*κ*B p65 was assessed by immunofluorescence staining. Scale bar = 20 *μ*m. Data are presented as mean ± SEM. ^∗^*P* < 0.05, ^∗∗^*P* < 0.01.

MyD88 is a critical regulator implicated in inflammation by modulating Nox2-dependent ROS production [15]. Moreover, ROS has been confirmed to activate NF-*κ*B pathway [28]. Herein, we investigate the effects of miR-30a on MyD88, Nox2, ROS, and NF-*κ*B. As illustrated in Figures 6(a) and 6(b), LTA notably induced the MyD88, Nox2, and p-p65 levels, accompanied by a noticeable increase in ROS generation; however, these values were decreased by the miR-30a overexpression. To verify whether the action of miR-30a is mediated by MyD88, we performed a rescue experiment by transfecting the cells with the recombinant plasmids overexpressing MyD88 (pcDNA3.1-MyD88). The results showed that the overexpression of MyD88 significantly alleviated LTA-induced MyD88, Nox2, p-p65, and ROS production (Figures 6(a) and 6(b)).Furthermore, upregulation of MyD88 also reversed the inhibitory effects of miR-30a on NF-*κ*B p65 (Figure 6(c)). These results demonstrate that miR-30a depresses oxidative stress and inflammatory responses by inhibiting the MyD88/Nox2/ROS/NF-*κ*B pathway.

### 3.7. Upregulation of miR-30a Attenuates LTA-Induced Endometritis in Mice

**Figure 7:**
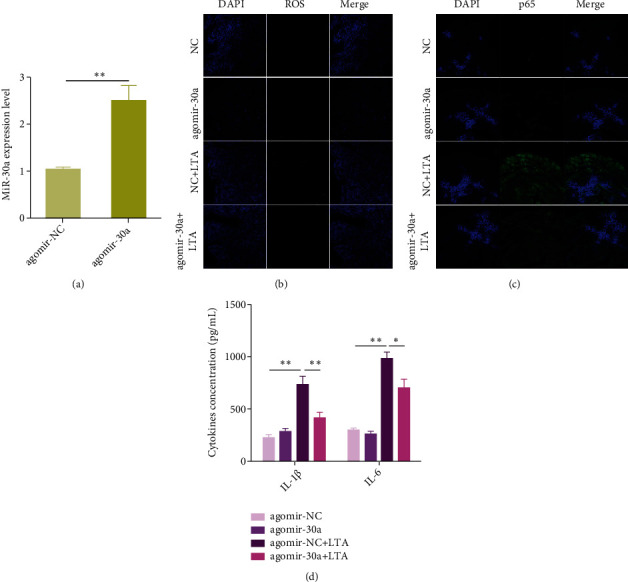
Upregulation of miR-30a improves LTA-induced endometritis in mice. (a) The mice were injected with chemically synthesized miR-30a agonists (agomir-30a), followed by intrauterine infusion of LTA to induce endometritis. The miR-30a expression was measured by qPCR. (b) Immunofluorescence staining of ROS. Scale bar = 50 *μ*m. (c) Immunofluorescence staining of NF-*κ*B p65. Scale bar = 50 *μ*m. (d) ELISA measurement of IL-1*β* and IL-6. Data are presented as mean ± SEM. ^∗^*P* < 0.05, ^∗∗^*P* < 0.01.

Our in vitro data revealed miR-30a functions as a key negative regulator of the MyD88/Nox2/ROS/NF-*κ*B pathway and thereby alleviates LTA-induced oxidative stress and inflammation. Next, to further evaluate the therapeutic potential of miR-30a in vivo, agomir-30a (miR-30a agonist) or agomir-NC (negative control) was utilized to transiently upregulate the miR-30a expression in mice, followed by infusion of LTA or PBS into the uterine cavity. As shown in Figure 7(a), administration with agomir-30a upregulated the miR-30a expression in uterine tissues when compared with agomir-NC. Furthermore, the miR-30a overexpression remarkably suppressed the accumulation of ROSinduced by LTA (Figure 7(b)), accompanied by reduction of p65 activation as well as IL-1*β* and IL-6 (Figures 7(c) and 7(d)). These data uncover the important role of miR-30a in endometritis and also provide a basis for developing novel approach to endometritis.

## 4. Discussion

Endometritis severely weakens the reproductive health of women and mammals and imposes a significant economic burden on dairy farms. Although many antibiotics such as oxytetracycline have been broadly employed for the control of endometritis, the abuse of antibiotics leads the emergence of drug-resistant strains in the environment. Hence, it is necessary to develop innovative treatment strategies for endometritis. Here, we verify that miR-30a exerts anti-inflammatory and antioxidant roles by inhibiting the MyD88/Nox2/ROS-activated NF-*κ*B pathway.

Microbial components evoke a wide range of intracellular signaling cascades that govern the host inflammatory reactions via activation of TLRs [29]. So far, 10 TLRs (TLR1-TLR10) have been clearly identified in cattle [30].TLR2 functions as the critical receptor for LTA from *S. aureus*. *S. aureus* LTA is able to act as an immune system stimulus, which plays a significant role in the regulation of many genes that are implicated in inflammatory diseases [8].A previous report has pointed that LTA evokes TNF-*α* release via the TLR2 signaling [12]. In general, theTLR2-mediated intracellular signaling is initiated by some important adapter proteins, such as MyD88. Upon activation of TLR2, MyD88 is recruited to TLR domains and then provokes downstream NF-*κ*B pathway [31]. Once NF-*κ*B is activated, one of its subunits, p65, transfers from cytoplasm to nucleus and then induces the transcription of inflammation-related genes, which ultimately leads to the development of endometritis [32]. Additionally, some proinflammatory cytokines including IL-1*β* and IL-6 also can promote the release of other inflammatory mediators, leading to apoptosis of epithelial cells, thereby aggravating endometrial damage. We found that the IL-1*β* and IL-6 levels were significantly elevated in uterine tissues with endometritis. In addition, their levels were also downregulated in LTA-stimulated BEND cells. Thus, suppression of the NF-*κ*B pathway can improve the outcome of some inflammatory diseases such as endometritis.

Over the last ten years, the regulatory function of miRNAs in inflammation has already received growing attention. Some miRNAs are able to be negative feedback modulators of inflammatory reactions by targeting proteins implicated in signal transduction [22, 33]. More importantly, a considerable number of miRNAs, includingmiR-30, have been found to be associated with endometritis [23]. miR-30a's nucleic acid sequence is highly conserved in cattle, human, and mice. It has been reported that miR-30a is involved in the process of tumor growth as a tumor inhibitor. For instance, the ectopic expression of miR-30a suppresses the proliferation and migration and tumorigenesis of gastric cancer cells by directly inhibiting ITGA2 [34].More notably, the immunomodulatory effect of miR-30a has been disclosed in several studies. Silence of miRNA-30a polarizes macrophages toward M2 phenotype to ameliorate cardiac injury through targeting SOCS1 [24]. Moreover, another study clearly showed that the overexpression of miR-30a in monocyte cells significantly attenuates *Mycobacterium tuberculosis* infection, as well as the secretion of TNF-*α*, IL-6, and IL-8 [35]. However, the underlying regulatory mechanisms of miR-30a in endometritis remain unclear. Here, the miR-30a expression was notably upregulated in endometritis tissue samples, suggesting its involvement in the development of endometritis. Previous studies have revealed that transactivation of miR-30b gene is dependent on NF-*κ*B p65 activation following *Cryptosporidium parvum* infection [27]. Therefore, we speculate that miR-30a, like miR-30b, is also regulated by NF-*κ*B. Here, we confirmed that LTA markedly induced the miR-30a expression, which was regulated by NF-*κ*B p65. Furthermore, analysis of the biological function of miR-30a revealed that the miR-30a overexpression decreased proinflammatory cytokine production and ROS accumulation, hinting that it possesses anti-inflammatory and antioxidant effects. Thus, the induction of miR-30a by LTA is considered to be an adaptive mechanism to protect the body from inflammatory damage. To further delineate the mechanism of miR-30a's anti-inflammatory effect, we predicted its molecular targets. Through an online prediction program, we screened out MyD88, an essential adaptor protein of TLR2 signaling, and further confirmed MyD88 as the target of miR-30a.

ROS are essential for the clearance of invading microorganisms, but their overproduction can induce inflammatory damage to tissues [13].The NADPH oxidase (Nox) family has merged as a major source of ROS in signal transduction [36]. ROS have been found to be involved in numerous inflammatory disorders such as atherosclerosis [37] and neurodegeneration [38]. Importantly, our studies have attributed the cellular damage in the pathologies of endometritis to oxidative stress. Nox2 is an important NADPH oxidase, and its activation is required for LTA-induced ROS production in brain astrocytes [39]. Furthermore, several lines of evidence have revealed that the recruitment of MyD88 to TLR2 leads to the activation of Nox2 and ROS generation [15]. Herein, our data demonstrated that the overexpression of miR-30a not only inhibited LTA-upregulated MyD88 but also decreased the Nox2expression, implying that miR-30a attenuates ROS generation via targeting MyD88/Nox2. Excessive oxidative stress during inflammatory injuries can shape gene expression patterns by a variety of transcription factors. It is worth noting that the increase of ROS level caused by external stimulation promotes the secretion of several inflammatory mediators [40]. Here, we investigated the roles of NF-*κ*B, which is widely recognized to regulate the process of oxidative stress-related inflammatory diseases. Our results showed that miR-30a inhibited the activities of NF-*κ*B p65. Indeed, ROS has been found to trigger the nuclear translocation of NF-*κ*B p65 [28].To further verify whether MyD88 mediates the anti-inflammatory and antioxidant properties of miR-30a, we then overexpressed the MyD88 gene expression in the presence of miR-30a mimics. In this report, the overexpression of MyD88 almost completely abolished miR-30a-decreased MyD8, ROS, and p-p65, suggesting that the biological functions of miR-30a were mediated by MyD88. Mice are commonly employed as the experimental animal models of various inflammatory diseases [10, 17]. Notably, a mouse model of LTA-induced endometritis has been confirmed to be a suitable animal model for exploring the potential therapeutic strategies against endometritis in cow [14].Herein, we further confirm the anti-inflammatory property of miR-30a in a murine model of endometritis. Consistent with the in vitro data, upregulation of miR-30a inhibited the inflammatory responses in uterine tissues of mice with endometritis.

## 5. Conclusion

**Figure 8:**
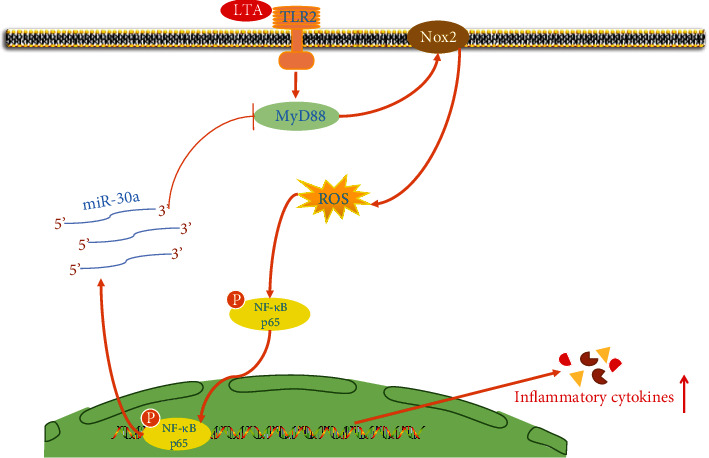
Schematic diagram of signalings related to anti-inflammatory and antioxidative effects of miR-30a on LTA-induced inflammatory responses.

Collectively, we provide the first evidence that upregulation of miR-30a curbs *S. aureus* LTA-triggered oxidative stress and inflammation via the suppression of the MyD88/Nox2/ROS/NF-*κ*B signaling (Figure 8). This study opens new avenues to explore miR-30a-based therapeutics against inflammatory diseases caused by *S. aureus* infection.

## Data Availability

The data used to support the findings of this study are available from the corresponding author upon reasonable request.

## Supplementary Materials

Supplementary Materials Supplementary Table 1: primer sequence for qPCR.
