# Supplementary material for: Therapeutic Role of miR-30a in Lipoteichoic Acid-Induced Endometritis via Targeting the MyD88/Nox2/ROS Signaling
Source: Oxid Med Cell Longev. 2021 Dec 31;2021:5042048. doi: 10.1155/2021/5042048 (PMC8741357; doi:10.1155/2021/5042048)
Supplement: Supplementary Materials — Supplementary Table 1: primer sequence for qPCR. [file 5042048.f1.docx]

**Supplementary Table 1.** Primer sequence for qPCR

| **Gene** | **Primer sequence (5'-3')** |
| --- | --- |
| IL-1β | F: AAAAATCCCTGGTGCTGGCT |
|  | R: GGGTGGGCGTATCACCTTTT |
| IL-6 | F: CTACCTCCAGAACGAGTATG |
|  | R: CAGCAGGTCAGTGTTTGTGG |
| GAPDH | F: GGTCACCAGGGCTGCTTT |
|  | R: CTGTGCCGTTGAACTTGC |
| bta-miR-30a | RT:GTCGTATCCAGTGCAGGGTCCGAGGTATTCGCACTGGATACGACAGCTTC |
|  | F: GCGTGTAAACATCCTCGACTG |
|  | R: AGTGCAGGGTCCGAGGTATT |
| bta-U6 | RT: ACGCTTCACGAATTTGCGTGTC |
|  | F: CTCGCTTCGGCAGCACATATACT |
|  | R: ACGCTTCACGAATTTGCGTGTC |
| mmu-miR-30a | RT:GTCGTATCCAGTGCAGGGTCCGAGGTATTCGCACTGGATACGACCTTCCA |
|  | F: CGCGTGTAAACATCCTCGAC |
|  | R: AGTGCAGGGTCCGAGGTATT |
| mmu-U6 | RT: ACGCTTCACGAATTTGCGTGTC |
|  | F: CTCGCTTCGGCAGCACATATACT |
|  | R: ACGCTTCACGAATTTGCGTGTC |
